# Risk of cervical precancer among HPV–negative women in the Netherlands and its association with previous HPV and cytology results: A follow-up analysis of a randomized screening study

**DOI:** 10.1371/journal.pmed.1004115

**Published:** 2022-10-28

**Authors:** Federica Inturrisi, Lawrence Rozendaal, Nienke J. Veldhuijzen, Daniëlle A. M. Heideman, Chris J. L. M. Meijer, Johannes Berkhof

**Affiliations:** 1 Amsterdam UMC location Vrije Universiteit Amsterdam, Epidemiology and Data Science, Amsterdam, the Netherlands; 2 Amsterdam Public Health, Methodology, Amsterdam, the Netherlands; 3 Amsterdam UMC location Vrije Universiteit Amsterdam, Pathology, Amsterdam, the Netherlands; 4 Cancer Center Amsterdam, Imaging and Biomarkers, Amsterdam, the Netherlands; Vanderbilt University School of Medicine, UNITED STATES

## Abstract

**Background:**

Human papillomavirus (HPV)-based screening programs still use one-size-fits-all protocols but efficiency and efficacy of programs may be improved by stratifying women based on previous screening results.

**Methods and findings:**

We studied the association between cervical intraepithelial neoplasia grade 3 or cancer (CIN3+) and previous screening results in the Population-Based Screening Study Amsterdam (POBASCAM) trial, performed in the Netherlands in the setting of regular screening, where women aged from 29 to 61 years old were invited to cytology and HPV co-testing at enrolment in year 1999/2002 and at the next round in 2003/2007. We selected 18,448 women (9,293 from the intervention group and 9,155 from the control group) who tested HPV–negative in 2003/2007 and did not have cervical intraepithelial neoplasia grade 2 or worse (CIN2+) or hysterectomy after enrolment. Follow-up was collected until 14 years after the 2003/2007 screen, covering 4 rounds of screening. Risk of CIN3+ and CIN2+ among women with an HPV–negative test, irrespective of previous round results and stratified according to previous round HPV and cytology results, were calculated by the Kaplan–Meier method.

During 14 years of follow-up, 62 CIN3+ cases (24 in the intervention group and 38 in the control group) were detected. HPV–negative women had a 14-year CIN3+ risk of 0.48% (95% confidence interval 0.37 to 0.62) and CIN2+ risk of 1.17% (0.99 to 1.38). The CIN3+ risk among HPV–negative women was increased in women with a previous positive HPV test (2.36%, 1.20 to 4.63; *p* < 0.001) or co-test (1.68%, 0.87 to 3.20; *p* < 0.001) and, equivalently, decreased in women with a previous negative HPV test (0.43%, 0.33 to 0.57) or a negative co-test (0.43%, 0.33 to 0.57). The CIN3+ risk was not influenced by the previous cytology result. The CIN3+ risk among HPV–negative women was increased after both a previous HPV16–positive test (3.90%, 1.47 to 10.12; *p* < 0.001) and a previous HPV16–negative/HPVother–positive test (1.91%, 0.76 to 4.74; *p* = 0.002). For endpoint CIN2+ (147 cases), findings were similar except that the CIN2+ risk was increased after previous abnormal cytology (4.06%, 2.30 to 7.12; *p* < 0.001). The presented risk estimates were calculated by tracking histological results through the Dutch nationwide pathology archive (PALGA) and were not adjusted for non-compliance with the colposcopy referral advice.

**Conclusions:**

HPV–negative women had an increased long-term risk of CIN3+ when the HPV test in the previous screening round was positive. This supports the implementation of risk-based intervals that depend on HPV results in the current and previous screening round.

**Trial registration:**

POBASCAM trial, trial registration number ISRCTN20781131.

## Abstract 2

Federica Inturrisi and colleagues investigate the risk of cervical precancer and its association with previous HPV and cytology results among HPV-negative women in the Netherlands.

## Author summary

**Why was this study done?:**

- HPV-based screening is being implemented in many countries worldwide in accordance with the WHO recommendation to use HPV testing as the primary screening method for prevention of cervical cancer.
- In the Netherlands, HPV-based screening was implemented in 2017 with a 5-year interval among screen-positive women aged from 30 to 60 years old and an extended 10-year interval among screen-negative women aged 40 or 50 years old.
- The safety of a 10-year interval for women who are HPV–negative at age 40 or 50 but who were HPV–positive in the previous screening round should be assessed.
- Efficiency and efficacy of HPV-based screening programs may be improved by stratifying women based on previous screening results.

**What did the researcher do and find?:**

- We collected follow-up data of women who participated in the Population-Based Screening Study Amsterdam (POBASCAM) trial with enrollment in 1999/2002 and 19 years of follow-up, selected 18,448 women with a negative HPV result at the second study-related screening round (5 years after enrolment) and stratified them according to screening results at the time of study enrolment.
- We estimated the 14-year stratum-specific risk of CIN3+ and CIN2+, covering 3 rounds of screening.
- The 14-year CIN3+ risk among HPV–negative women was 0.48%, was slightly lower when the HPV test in the previous round was negative (0.43%), but was markedly higher when the HPV test in the previous round was positive (2.36%).
- The 14-year CIN3+ risk among HPV–negative women was not increased when the cytology test in the previous round was positive.

**What do these findings mean?:**

- Our results confirmed the safety of extending the screening interval from 5 to 10 years when switching from cytology-based screening to HPV-based screening.
- A screening extension from 5 to 10 years can be maintained at the second HPV-based screening round when the HPV result in the previous round was negative.
- A history of HPV positive screening results is associated with an increased future risk of precancer also when the current HPV result is negative.
- HPV results from multiple screening rounds should be considered when determining the time to the next screening invitation.

## Introduction

Human papillomavirus (HPV) causes virtually all cases of cervical cancer and its precursors. Testing for HPV DNA has demonstrated earlier detection of cervical intraepithelial neoplasia grade 3 or worse (CIN3+) and greater protection against invasive cervical cancer than cytology [1–3]. This has led to the revision of cervical cancer screening guidelines recommending HPV testing as single primary test in multiple European countries, New Zealand, Australia, and the United States [4–7].

HPV testing is less specific than cytological testing, but HPV-based screening programs may achieve a higher level of efficiency by extending the screening interval after a negative primary HPV test. The new Dutch HPV-based cervical screening program, which screens women aged from 30 to 60 years old every 5 years, was implemented in 2017 with an extension of the screening interval from 5 to 10 years for HPV–negative women aged 40 or 50 years old [8,9]. The question arises whether this extension is also tenable after 2 rounds of HPV-based screening. It will be especially critical to establish the safety of a 10-year interval for women who are HPV–negative at age 40 or 50 but who were HPV–positive in the previous screening round. There is only limited evidence on the future risk of cervical (pre)cancer by previous screening results. The main reason for this is that organized HPV-based screening programs have been implemented recently and most programs have not yet completed 2 rounds of HPV-based screening. Real-world evidence has been collected in the Kaiser Permanente Northern California (KPNC) cohort, initiated in 2003 with follow-up until 2015, where women were recommended 3-yearly HPV and cytology co-testing. In this cohort, it was found that the cancer and CIN3+ risks in HPV–negative women decreased with an increasing number of negative historical co-tests [10] and that a history of HPV–positive results increases risk, even when the current result is negative [11]. Risks by previous screening results beyond 5 years are yet to be studied.

In order to gain insight into the safety of HPV-based screening intervals longer than 5 years by previous screening results, we collected follow-up data from the POBASCAM (Population-Based Screening Study Amsterdam) trial. This HPV-based screening trial offers a unique opportunity to anticipate risk estimation based on longitudinal HPV-based screening data, because participating women received HPV testing in 2 consecutive screening rounds, the first at enrolment in 1999/2002 and the second 5 years later [1]. We extended the follow-up of POBASCAM women up to 14 years after the second HPV screen, covering 4 screening rounds in total. We analyzed data of women with an HPV–negative screening test at the second HPV-based screening round to determine the effect of the previous HPV test, cytology, and co-test result on the long-term risk of cervical (pre)cancer.

## Methods

### Study population

This is a longitudinal study in which we collected passive follow-up of women who participated in the POBASCAM trial. The POBASCAM trial (trial registration number ISRCTN20781131) was conducted in the setting of the regular (cytology) cervical cancer screening program in the Netherlands that invites women aged from 30 to 60 years old every 5 years. The design has been published previously [1,12,13]. In brief, the enrolment period was from January 1999 to September 2002. All women provided written informed consent prior enrolment. Eligible consenting women (*N* = 44,102) aged from 29 to 61 years old were randomly assigned (1:1) to receive HPV and cytology co-testing (intervention) or cytology-only with blinded HPV (control). In the intervention group, women with normal cytology and HPV–negative result were invited to the next screening round after 5 years. Women with high-grade abnormal cytology (HSIL) were referred for colposcopy irrespective of the HPV result. Women with borderline or low-grade cytology (ASCUS/LSIL) and those with normal cytology and HPV–positive result were advised to undergo repeat co-testing at 6 or 18 months, and referred for colposcopy in case of HSIL and/or HPV–positive result. Women without HSIL and HPV–negative result were invited at the next round after 5 years. In the control group, women with normal cytology were invited to the next round after 5 years. Women with HSIL cytology were referred for colposcopy. Women with ASCUS/LSIL were advised to repeat cytology after 6 or 18 months and referred for colposcopy in case the repeat cytology result was abnormal (ASCUS+). Women with normal repeat cytology were invited at the next round after 5 years. Post-colposcopy management was done by cytology at 6, 12, and possibly 24 months after colposcopy and, if no lesion was detected, women were referred back to routine screening. At the second screening round after 5 years, women in both study groups were managed according to the intervention protocol (HPV and cytology co-testing). At the third screening round after 10 years, women in both study groups were managed according to the control protocol (cytology-only), which was in line with the screening program at that time. At the fourth screening round after 15 years, women invited before January 2017 were managed according to the control protocol and women invited at a later time were managed according to the new primary HPV screening program. In the new program, HPV–negative women are invited to the next screening round after 5 to 10 years and HPV–positive women are tested by cytology. HPV–positive women with ASCUS+ at baseline are directly referred for colposcopy, while HPV–positive women with normal cytology are invited for repeat cytology testing after 6 months, and referred for colposcopy when ASCUS+. Women with normal repeat cytology are invited at the next round after 5 years.

In the POBASCAM trial, a conventional cervical smear test was prepared on a glass slide for cytology reading after which the brush was placed in a vial for HPV DNA testing done using high-risk HPV GP5+/6+ PCR-enzyme immunoassay (EIA) [14] that detects 14 high-risk HPV genotypes (types 16, 18, 31, 33, 35, 39, 45, 51, 52, 56, 58, 59, 66, and 68). EIA-positive specimens were considered “generic” high-risk HPV–positive and were genotyped by reverse line blotting [15]. Cytology and HPV testing were performed without knowledge of the other test result. In the new HPV-based screening program, the cervical sample was stored in 20-ml PreservCyt (Hologic, Marlborough, Massachusetts, United States of America) and HPV DNA testing was performed by Cobas 4800 HPV Test (Cobas 4800 System, Roche Molecular Systems, Branchburg, New Jersey, USA)[16]. Women who did not wish to have a cervical sample taken at their clinician’s office (Cervex Brush, Rovers Medical Devices B.V., Oss, NL) could request a self-sampling kit at home (Evalyn Brush, Rovers Medical Devices B.V., Oss, NL). The dry brush was sent to the laboratory, stored in 20-ml PreservCyt and HPV DNA testing was performed by Cobas 4800 HPV Test. In case the HPV self-sampling test was positive, a new sample was collected at the clinician’s office for cytological evaluation. When a woman was referred for colposcopy, suspected areas on the cervix were identified and biopsies were taken for histological examination. Histology was examined locally and classified as normal, cervical intraepithelial neoplasia (CIN) grade 1, 2, 3, or invasive cancer, according to international criteria [17]. Adenocarcinoma in situ was added to CIN3. Treatment by loop electrosurgical excision procedure was recommended to women diagnosed with CIN2 or worse (CIN2+). Follow-up histology was obtained through the PALGA nationwide histopathology and cytopathology registry [18] up to January 2018.

The POBASCAM trial was approved by the VU University Medical Centre (no 96/103) and the Ministry of Public Health (no 328650). This study was approved by the scientific committee of PALGA.

### Statistical analyses

We included women from the intervention and control group of the POBASCAM trial who were invited for co-testing and had a negative HPV test result at the second screening round. Hereby, we will refer to these women as HPV–negative women or as the total study sample. Those with a CIN2+ diagnosis or hysterectomy before the start of the second screening round were excluded. Follow-up started counting at the second screening round visit and ended on the date of a CIN3+ diagnosis or hysterectomy. As detection of CIN3+ after a positive screen can take up to 4 years (because of repeat testing at 6 and 18 months and post-colposcopy follow-up for up to 24 months; see above “Study population”), the 4-year cutoff corresponds with the risk of prevalent disease, the 9-year cutoff corresponds with the risk of CIN3+ because of a positive screen after 5 years, and the 14-year cutoff represents the risk of CIN3+ because of a positive screen after 10 years. If the second screening round was the last attended screening round and no CIN3+ or hysterectomy was reported, follow-up was censored 4 years after the starting date of the second round. If the third screening round was the last attended and no CIN3+ or hysterectomy was reported, follow-up was censored 9 years after the starting date of the second round. In all other women, follow-up ended 14 years after the start of the second round or on 31 January 2018, whichever came first. The approach for censoring the data was also used in previous follow-up analyses of the POBASCAM trial (e.g., [9]) and is in line with the intention-to-treat publications of the POBASCAM trial [1,12]. Notably, in the Dutch screening program, a new screening invitation is sent in the year a woman turns 30, 35, 40, 45, 50, 55, 60 and therefore cutoffs of 4, 9, and 14 years after the first screen to define subsequent screening rounds correspond with completion of the first, second, and third round, respectively.

We measured time to CIN3+ detection and used the Kaplan–Meier method to estimate the 14-year cumulative risk of CIN3+, stratified by previous test results. Previous test results were HPV test, cytology and co-test results obtained at the start of the first screening round, which corresponds to enrolment in the POBASCAM trial. Analyses were repeated using the last test of the first screening round (including repeat testing) as previous test result. HPV was labeled as positive if the result was positive at the generic HPV test and negative otherwise. Cytology was labeled as positive if the result was borderline or worse (ASCUS+) and negative otherwise. Co-test was labeled as positive if HPV and/or cytology were positive and negative if they were both negative.

We reported absolute risks with ninety-five percent confidence intervals (95% CI). We also reported risk differences (RDs) with 95% CI and *p*-values for the comparison of CIN3+ risks after a positive and negative screening result in the previous round. Separate risk comparisons were made for previous round HPV, cytology, and co-test results. The 95% CI were calculated by parametric bootstrapping and *p*-values were calculated by Wald testing, assuming a normal distribution for the logarithmic risk. Separate estimates were reported for intervention and control group, and for the pooled estimate over the 2 study groups. Finally, we calculated separate CIN3+ risks for age strata <39 years, 39 to 48 years, and ≥49 years at the second round, defined as matching with the screening invitations, and genotype results at the previous round, using hierarchical ranking based on HPV genotype groups HPV16, HPV18/45, HPV31/33/35/52/58, and other high-risk HPV genotypes. Statistical significance was considered at a significance level of 5%. All analyses were repeated for endpoint CIN2+ and the main analysis was repeated for endpoint cancer. All analyses were performed using Microsoft Excel 2016 and STATA/SE 14.1, except for bootstrapping and visualization that were performed using R version 4.0.3. We followed the STROBE guidelines for reporting of observational studies (S2 File).

## Results

**Fig 1:**
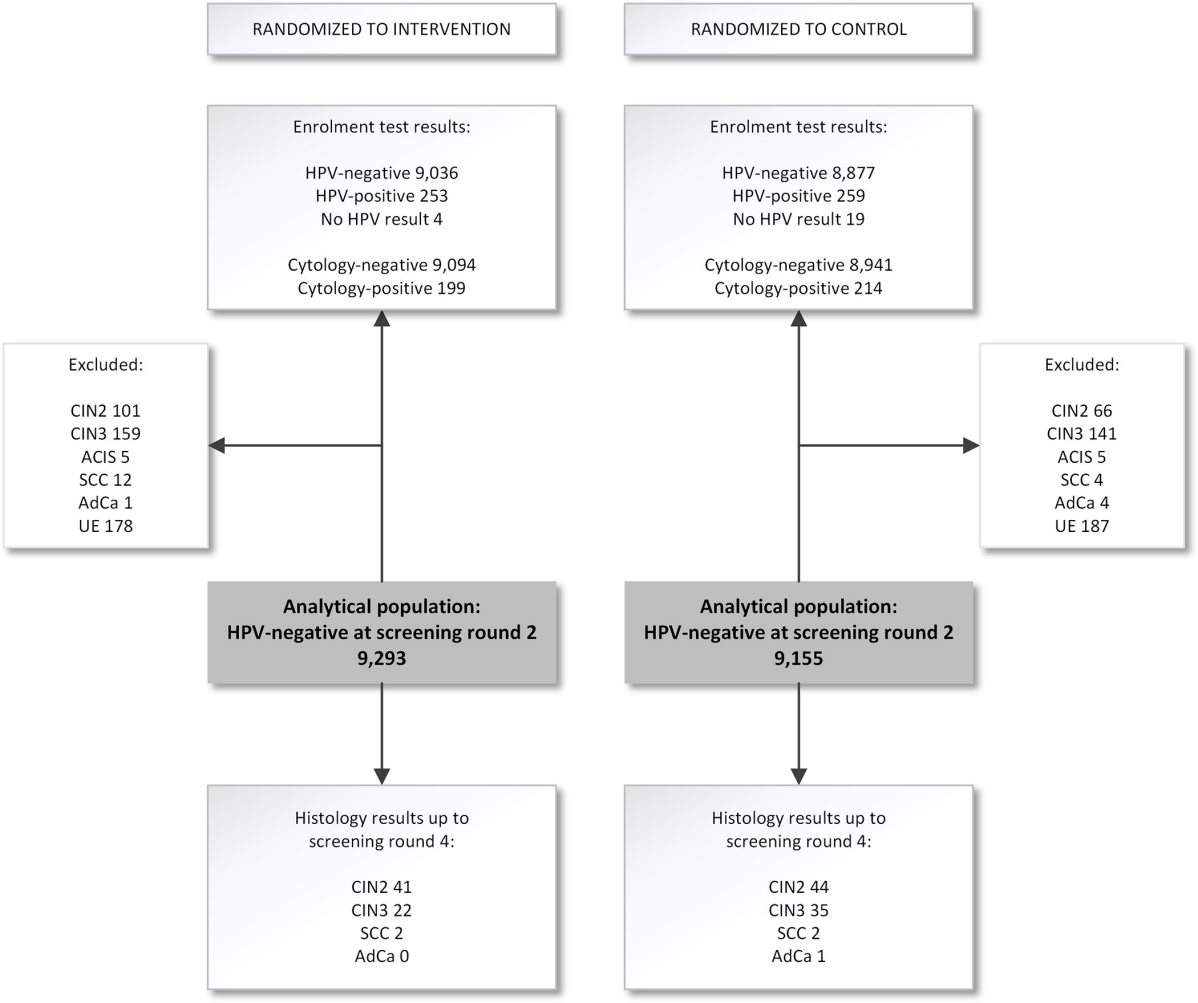
Flowchart of the 19 years of follow-up of the POBASCAM trial with focus on the analytical population consisting of 18,448 women who tested HPV–negative at the second screening round 5 years after enrolment. ACIS, adenocarcinoma in situ; AdCa, adenocarcinoma; CIN2/3, cervical intraepithelial neoplasia grade 2/3; HPV, human papillomavirus; SCC, squamous cell carcinoma; UE, uterus extirpation (hysterectomy).

The screening profile and characteristics of women who participated in the POBASCAM trial and had an HPV–negative study-related second screening test is presented in Fig 1 and Table 1. We included a total of 18,448 HPV–negative women, 9,293 from the intervention group and 9,155 from the control group. Their median age at the second screening round was 45 years (range 33 to 67) in both study groups (Table 1). During 14 years of follow-up, 41 CIN2, 22 CIN3, and 2 squamous cell carcinomas were detected in the intervention group; and 44 CIN2, 35 CIN3, 2 squamous cell carcinomas, and 1 adenocarcinoma were detected in the control group.

**Table 1:** Characteristics of the study sample.

	N	Age	Time since last test in previous round (years) *	CIN2	CIN3	Cancer
Total	18,448	45 (33 to 67)	−4.9 (−1.7 to −8.4)	85	57	5
POBASCAM study group						
Intervention	9,293	46 (33 to 67)	−4.9 (−1.7 to −8.4)	41	22	2
Control	9,155	45 (33 to 67)	−4.9 (−1.8 to −8.4)	44	35	3
HPV previous round						
HPV+	512	40 (33 to 65)	−4.4 (−1.9 to −6.9)	11	7	2
HPV−	17,913	46 (33 to 67)	−4.9 (−1.7 to −8.4)	74	50	3
HPV missing	23					
Cytology previous round						
Cytology+	413	45 (33 to 67)	−3.9 (−1.7 to −6.8)	12	1	0
Cytology−	18,035	45 (33 to 67)	−4.9 (−1.8 to −8.4)	73	56	5
Co-test previous round						
Co-test+	834	44 (33 to 65)	−4.2 (−1.7 to −6.9)	18	8	2
Co-test−	17,591	46 (33 to 67)	−4.9 (−1.8 to −8.4)	67	49	3
Co-test missing	23					

* Time to HPV–negative test result since latest test (e.g., screening invitation test, a repeat test, or a post-colposcopy follow-up test) in the previous screening round.

CIN2/3, cervical intraepithelial neoplasia grade 2/3; HPV, human papillomavirus.

**Fig 2:**
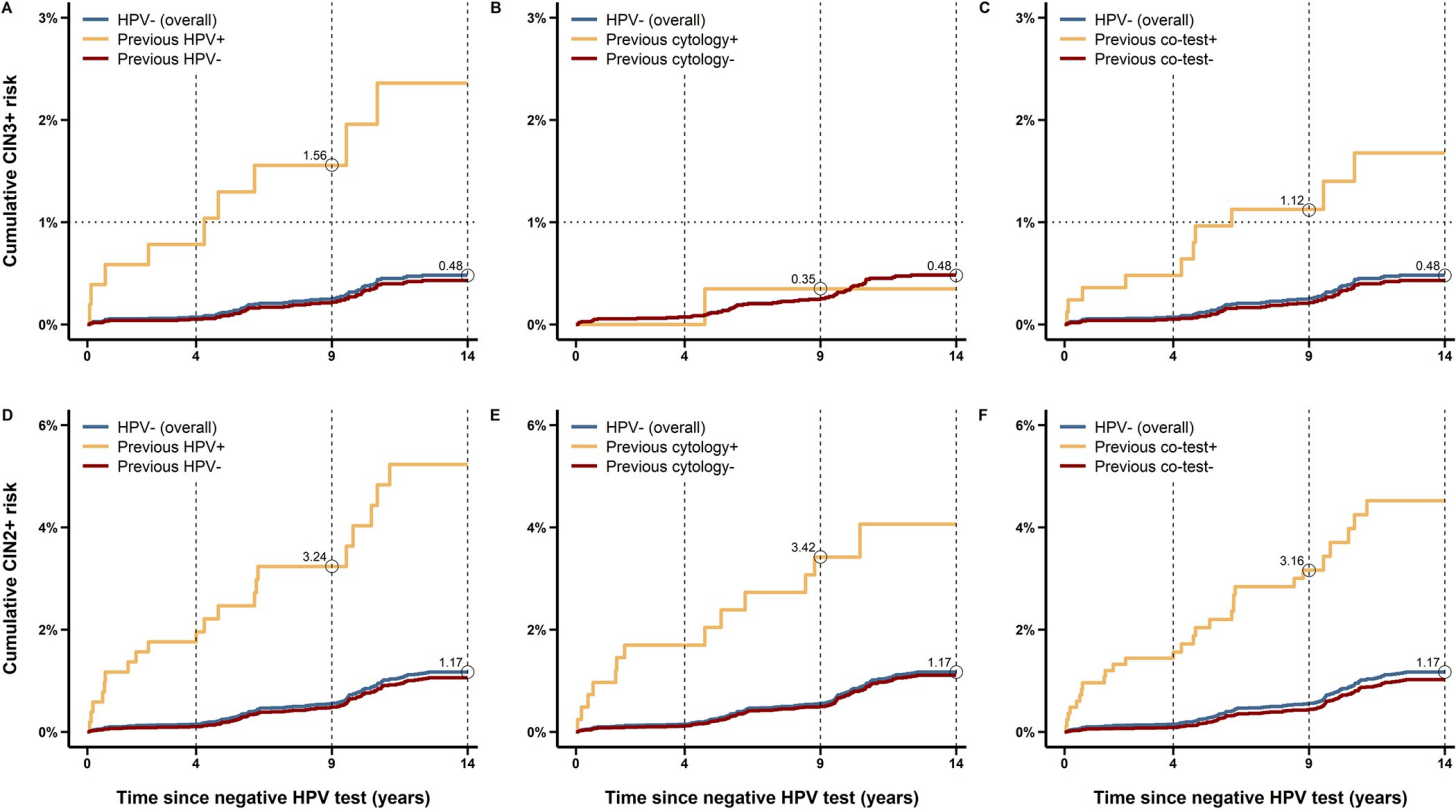
Absolute risks of CIN3+ and CIN2+ among HPV–negative women (pooled study groups) stratified for previous test result, after up to 3 screening rounds (14 years). Panels (A) to (C) refer to endpoint CIN3+ ((A) by previous HPV test, (B) by previous cytology test, (C) by previous co-test) and include a dotted line at 1% CIN3+ corresponding to the informal Dutch threshold for the next screening time point; panels (D) to (F) refer to endpoint CIN2+ ((D) by previous HPV test, (E) by previous cytology test, (F) by previous co-test). Values embedded in the plots represent the 14-year risk among HPV–negative women (overall; blue line) and the 9-year risk among HPV–negative women with a previous positive test (yellow line). In panel (B), the blue line is not visible due to the overlap with the red line. CIN2/3+, cervical intraepithelial neoplasia grade 2/3 or worse; HPV, human papillomavirus.

Women with an HPV–negative result had a 14-year cumulative CIN3+ risk of 0.48% (95% CI 0.37 to 0.62) (Table 2 and Fig 2). A markedly increased CIN3+ risk was observed among HPV–negative women who in the previous round had a positive HPV test (2.36%, 95% CI 1.20 to 4.63; *p* < 0.001) or a positive co-test (1.68%, 95% CI 0.87 to 3.20; *p* < 0.001). Equivalently, a decreased CIN3+ risk was observed among HPV–negative women who in the previous round also had a negative HPV test (0.43%, 95% CI 0.33 to 0.57) or a negative co-test (0.43%, 95% CI 0.33 to 0.57). We did not find an increased CIN3+ risk in HPV–negative women who in the previous round had a positive cytology test (0.35%, 95% CI 0.05 to 2.45; *p* = 0.754) or, equivalently, not a significantly decreased CIN3+ risk in those who in previous round had a negative cytology test (0.48%, 95% CI 0.37 to 0.63). Intervention and control group-specific estimates of the absolute CIN3+ risks were similar despite the different screening management in the first round after enrolment (Table 2 and Figs A and B in S1 File). The 14-year CIN3+ risks among HPV–negative women were 0.39% (95% CI 0.26 to 0.59) in the intervention group and 0.57% (95% CI 0.41 to 0.79) in the control group. Increased CIN3+ risks were found among HPV–negative with a previous positive HPV test (intervention group: 2.38%, 95% CI 1.13 to 6.97; *p* < 0.001; control group: 1.98%, 95% CI 0.72 to 5.38; *p* = 0.015) or a previous positive co-test (intervention group: 1.77%, 95% CI 0.70 to 4.43; *p* = 0.001; control group: 1.60%, 95% CI 0.64 to 3.97; *p* = 0.027).

**Table 2:** Absolute risks of CIN3+ and CIN2+ and risk differences at 14-year among HPV–negative women stratified for previous test result and study group.

		CIN3+				CIN2+			
	N	N events	Risk % (95% CI)	RD % (95% CI)	*p*-value	N events	Risk % (95% CI)	RD % (95% CI)	*p*-value
*Pooled study groups*									
Total	18,448	62	0.48 (0.37 to 0.62)			147	1.17 (0.99 to 1.38)		
*Previous round*									
HPV+	512	9	2.36 (1.20 to 4.63)	1.93 (0.76 to 4.19)	<0.001	20	5.23 (3.33 to 8.18)	4.17 (2.26 to 7.14)	<0.001
HPV−	17,913	53	0.43 (0.33 to 0.57)			127	1.06 (0.89 to 1.26)		
Cytology+	413	1	0.35 (0.05 to 2.45)	−0.13 (−0.47 to 2.00)	0.754	13	4.06 (2.30 to 7.12)	2.95 (1.18 to 6.03)	<0.001
Cytology−	18,035	61	0.48 (0.37 to 0.63)			134	1.11 (0.93 to 1.32)		
Co-test+	834	10	1.68 (0.87 to 3.20)	1.25 (0.43 to 2.80)	<0.001	28	4.52 (3.08 to 6.62)	3.50 (2.05 to 5.60)	<0.001
Co-test−	17,591	52	0.43 (0.33 to 0.57)			119	1.02 (0.85 to 1.23)		
*Intervention group*									
Total	9,293	24	0.39 (0.26 to 0.59)			65	1.05 (0.82 to 1.35)		
*Previous round*									
HPV+	253	5	2.82 (1.13 to 6.97)	2.49 (0.78 to 6.67)	<0.001	12	6.73 (3.73 to 11.99)	5.83 (2.83 to 11.18)	<0.001
HPV−	9,036	19	0.33 (0.21 to 0.52)			53	0.90 (0.69 to 1.19)		
Cytology+	199	0	0.0	-	-	8	5.32 (2.54 to 10.95)	4.36 (1.57 to 10.11)	<0.001
Cytology−	9,094	24	0.40 (0.26 to 0.60)			57	0.96 (0.74 to 1.26)		
Co-test+	413	5	1.77 (0.70 to 4.43)	1.44 (0.35 to 4.10)	0.001	16	5.38 (3.22 to 8.94)	4.51 (2.32 to 8.13)	<0.001
Co-test−	8,876	19	0.33 (0.21 to 0.53)			49	0.87 (0.65 to 1.15)		
*Control group*									
Total	9,155	38	0.57 (0.41 to 0.79)			82	1.29 (1.03 to 1.61)		
*Previous round*									
HPV+	259	4	1.98 (0.72 to 5.38)	1.45 (0.17 to 4.91)	0.015	8	3.94 (1.94 to 7.94)	2.73 (0.70 to 6.78)	0.002
HPV−	8,877	34	0.53 (0.38 to 0.75)			74	1.21 (0.96 to 1.53)		
Cytology+	214	1	0.65 (0.09 to 4.52)	0.08 (−0.55 to 4.07)	0.897	5	2.88 (1.19 to 6.85)	1.63 (−0.09 to 5.63)	0.070
Cytology−	8,941	37	0.57 (0.41 to 0.79)			77	1.25 (1.00 to 1.57)		
Co-test+	421	5	1.60 (0.64 to 3.97)	1.07 (0.08 to 3.47)	0.027	12	3.73 (2.08 to 6.64)	2.55 (0.86 to 5.51)	<0.001
Co-test−	8,715	33	0.53 (0.37 to 0.75)			70	1.18 (0.93 to 1.50)		

CI, confidence interval; CIN2/3+, cervical intraepithelial neoplasia grade 2/3 or worse; HPV, human papillomavirus; RD, risk difference.

In second-round age strata <39 years, 39 to 48 years, and ≥49 years, the 14-year CIN3+ risks were 0.95% (95% CI 0.60 to 1.51), 0.54% (95% CI 0.39 to 0.74), and 0.17% (95% CI 0.07 to 0.40) (Table A in S1 File). The direction of the effects of the previous screening test(s) on CIN3+ were the same for all age subgroups, although most pronounced for women with age 39 to 48.

The 14-year CIN3+ risk among HPV–negative women was increased in all genotype-specific subgroups with a previous round positive HPV test: HPV16–positive (3.90%, 95% CI 1.47 to 10.12; *p* < 0.001) and HPV16–negative/HPVother–positive (1.91%, 95% CI 0.76 to 4.74; *p* = 0.002). The CIN3+ risk was also increased among women who, in the previous round, were HPV31/33/35/52/58–positive but HPV16/18/45–negative (2.32%, 95% CI 0.56 to 9.25; *p* = 0.021), or positive for high-risk HPV genotypes other than HPV16/18/45/31/33/35/52/58 (2.06%, 95% CI 0.48 to 8.62; *p* = 0.037). The CIN3+ risk was not significantly increased among women who, in the previous round, tested HPV18/45–positive but HPV16–negative (1.10%, 95% CI 0.16 to 7.54; *p* = 0.348).

The analyses were repeated for endpoint CIN2+ and similar estimates were found when stratifying according to HPV and co-test results in the previous round (Table 2 and Fig 2). However, among HPV–negative women the CIN2+ risk (1.17%, 95% CI 0.99 to 1.38) was increased after a positive cytology test in the previous round (4.06%, 95% CI 2.30 to 7.12; *p* < 0.001) and, equivalently, decreased after a negative cytology test in the previous round (1.11%, 95% CI 0.93 to 1.32). The 14-year CIN2+ risk was increased in all genotype-specific subgroups with a previous round positive HPV test, including the HPV18/45–positive but HPV16–negative subgroup.

Similar estimates were found for endpoint cancer. The overall 14-year absolute risk of cancer was 0.03% (95% CI 0.01 to 0.08). The 14-year cancer risk was increased in HPV–negative women with a previous positive HPV test (0.52%, 95% CI 0.13 to 2.07; *p* < 0.001) or a previous positive co-test (0.32%, 95% CI 0.08 to 1.29; *p* = 0.002), and decreased in those with a previous negative HPV test (0.02%, 95% CI 0.01 to 0.06) or a previous negative co-test (0.02%, 95% CI 0.01 to 0.06). In our sample, there were no cancer cases among HPV–negative women with a previous positive cytology test.

Results were similar when the analysis was based on the last preceding test result instead of the first screening test result of the previous screening round (Table B in S1 File). An analysis of all women, including those with CIN2+ before the start of the second round, did not yield different results because only 40 of the 494 women with CIN2+ in the first round had a negative HPV result at the second screening round and only 1 of them developed CIN2 during 14 years of follow-up.

## Discussion

We evaluated the long-term safety of a negative HPV test result, expressed as 14-year risks of CIN3+ and CIN2+, stratified according to previous round screening outcomes. We found that the long-term risk of CIN3+ after a negative HPV test is very low when women had a negative HPV test result in the previous screening round. However, HPV–negative women who had a previous positive HPV test had a markedly increased risk of CIN3+. A previous abnormal cytology result gave an increased risk of CIN2+ but was not associated with an increased CIN3+ risk. Altogether, our results indicate that HPV results from multiple screening rounds should be considered when determining the time to the next screening invitation.

The major strengths of the current study are its large size, the long follow-up of 19 years after study enrolment, and the fact that the study was nested within a randomized population-based screening trial. This means that all women in our study had 2 rounds of HPV testing so that our study has both a high internal validity and is representative of an HPV screening setting. Risk estimates were not influenced by post-colposcopy follow-up because all women in our study population were invited for the second HPV-based screening round of the POBASCAM study. Besides, in women with a colposcopy referral in the first round, post-colposcopy management was completed about 2 years before the second round screening invitation was received. Risk estimates were similar in the intervention and control study group, indicating that the management of screen-positives in the previous round had only a limited effect on the CIN3+ and CIN2+ risks after a negative HPV test, if at all. A limitation to our study was that the presented CIN3+ and CIN2+ risk estimates were calculated by tracking histological results through the nationwide histopathology and cytopathology registry PALGA that does not contain information on gynecological procedures. That means that we were not able to assess how many cases were missed because women did not comply with the colposcopy referral advice. Furthermore, the national screening protocol varied over the course of the follow-up with cytology testing at the third screen after start of the POBASCAM study and primary testing by HPV alone at the fourth screen. This may have an effect on the absolute CIN3+ and CIN2+ risks, although we expect the effect on the relative risks to be small. A third limitation is that our study does not explain why a positive HPV result in the previous round leads to an increased long-term CIN3+ and CIN2+ risk in HPV–negative women. To study this, more information would be needed on the molecular features of the HPV-initiated lesions detected during follow-up. Several cohort studies with long-term follow-up indicated that risk of HPV infection and cervical lesions may be increased in women with previous HPV exposure because of person-specific variation in HPV susceptibility and in HPV clearance and because of viral re-activation [19–22]. Besides, it cannot be ruled out that some of the HPV–negative women had a false negative HPV test result.

This study builds on a small body of literature on the long-term risk among screen-negative women in the context of HPV-based screening. Our absolute risk estimates in HPV–negative women are in concordance with other studies showing that a negative HPV test result is associated with a low long-term risk of cervical (pre)cancer [1–3,16,23–27]. A previous post-hoc analysis of the POBASCAM cohort with 13 years of follow-up [9] showed a CIN3+ risk after a negative HPV result slightly higher than ours (0.56% versus 0.48%), but women were on average 5 years younger. On the basis of that long-term risk analysis [9] and supported by modeling studies [28,29], a risk-based extension was implemented in the Dutch HPV program in 2017 by extending the screening interval from 5 to 10 years for women aged 40 or 50 years old with a negative HPV test. Our study showed that a more precise risk assessment is possible when taking into account information of the previous screening round. The absolute CIN3+ risk after negative HPV results in both current and previous round is similar to that after an HPV–negative result in the current round. This means that a previous negative HPV result is not likely to change recommendation. However, the CIN3+ risk among HPV–negative women increased from 0.48% to 2.36% when the previous round HPV result was positive. This translates into a 10-year CIN3+ risk above the 1% CIN3+ risk that is currently used as an informal threshold for the next screening time point. Therefore, a previous positive HPV result asks for prudence and retesting after shorter interval may be needed. To our knowledge, the KPNC cohort is the only other large cohort with follow-up after a negative HPV test in the second HPV-based screening round. The change in CIN3+ risk that we observed when adding a previous round negative HPV test to the current negative HPV test was similar to that in the KPNC study reported by Castle and colleagues (2018) [10] and Egemen and colleagues (2020) [11], but our absolute CIN3+ risks were substantially higher. Besides, we reported a larger risk difference in case of a previous round positive HPV test than Egemen [11]. The high absolute risks observed in our study may be related to the length of the screening interval, which is 5 years in our study and 3 years in KPNC, and we also used a longer follow-up time in our analyses. Besides, 99.9% of the women in our study and 60% of the women in KPNC had a previous co-test result. This may influence the change in CIN3+ risk obtained when adding a previous round test result, because non-compliance to screening guidelines is a main risk factor of CIN3+ and cervical cancer [30,31]. Therefore, our study builds on the KPNC findings by estimating longer (14-year) CIN3+ risk among HPV–negative women in the setting of a randomized HPV-based screening trial with a 5-year screening interval.

Organized screening programs have nowadays linked digitalized screening registries and invitation systems [32], meaning that risk factors could readily be used for risk stratification. Such a digitalized registry has made it possible to implement a risk-based HPV program in the Netherlands, with an extended 10-year screening interval for HPV–negative women above age 40. Our study showed that, after 2 rounds of HPV screening, the safety of an extended screening interval is confirmed only among women who had a negative HPV test at the previous round. Women who had a positive HPV test at the previous round may benefit from a shorter screening interval such as a 5-year interval. Since the use of different intervals for HPV–negative women adds to the complexity of organized screening programs, it needs to be assessed whether inclusion of previous round results is feasible and cost-effective. Such an evaluation is urgently needed for countries like the Netherlands that have already implemented primary HPV screening and where the first women will have their second HPV screen in 2022.

Our study confirmed the safety of extending the screening interval from 5 to 10 years among HPV–negative women who were also screen-negative at the previous round. A history of HPV positivity, even when the current result is negative, is associated with a long-term increased precancer risk that warrants a re-evaluation of the extended 10-year screening interval. Altogether, our results indicate that HPV results from multiple screening rounds should be considered when determining the time to the next screening invitation.

## Supporting information

S1 File Supplementary Appendix. (DOCX)

S2 File STROBE Checklist. (DOCX)

## Abbreviations

ASCUS: atypical squamous cells of undetermined significance
CI: confidence interval
CIN(2/3+): cervical intraepithelial neoplasia (grade 2/3 or worse
EIA: enzyme immunoassay
HPV: human papillomavirus
GP5+/6+ PCR-EIA: general primer polymerase chain reaction-enzyme immunoassay
HSIL: high-grade squamous intraepithelial lesion
KPNC: Kaiser Permanente Northern California
LSIL: low-grade squamous intraepithelial lesion
PALGA: Dutch nationwide registry of histopathology and cytopathology
POBASCAM: Population-Based Screening Study Amsterdam
RD: risk difference
