# Supplementary material for: Risk of cervical precancer among HPV–negative women in the Netherlands and its association with previous HPV and cytology results: A follow-up analysis of a randomized screening study
Source: PLoS Med. 2022 Oct 28;19(10):e1004115. doi: 10.1371/journal.pmed.1004115 (PMC9616214; doi:10.1371/journal.pmed.1004115)
Supplement: S1 File — (DOCX) [file pmed.1004115.s001.docx]

**S1 File: Supplementary Appendix**

Supplement to:

**Risk of cervical precancer among HPV-negative women in the Netherlands and its association with previous HPV and cytology results: a follow-up analysis of a randomized screening study**

Federica Inturrisi, Lawrence Rozendaal, Nienke J. Veldhuijzen, Daniëlle A.M. Heideman, Chris J.L.M. Meijer, Johannes Berkhof

**Table of Contents**

[**Supplementary Figures** 2](#_Toc117173511)

[Fig A. Absolute risks of CIN3+ and CIN2+ amongst HPV-negative women randomized to the intervention trial group stratified for previous test result, after up to 3 screening rounds (14 years). 2](#_Toc117173512)

[Fig B. Absolute risks of CIN3+ and CIN2+ amongst HPV-negative women randomized to the control trial group stratified for previous test result, after up to 3 screening rounds (14 years). 3](#_Toc117173513)

[**Supplementary Tables** 4](#_Toc117173514)

[Table A. Absolute risks of CIN3+ and CIN2+ at 14-year amongst HPV-negative women stratified for previous test result and age. 4](#_Toc117173515)

[Table B. Absolute risks of CIN3+ and CIN2+ at 14-year amongst HPV-negative women stratified for previous test result, when the last preceding test result is used for stratification (sensitivity analysis). 6](#_Toc117173516)

# **Supplementary Figures**

## **Fig A. Absolute risks of CIN3+ and CIN2+ amongst HPV-negative women randomized to the intervention trial group stratified for previous test result, after up to 3 screening rounds (14 years).**

Panels (A) to (C) refer to endpoint CIN3+ ((A) by previous HPV test, (B) by previous cytology test, (C) by previous co-test) and include a dotted line at 1% CIN3+ corresponding to the informal Dutch threshold for the next screening time-point; panels (D) to (F) refer to endpoint CIN2+ ((D) by previous HPV test, (E) by previous cytology test, (F) by previous co-test). Values embedded in the plots represent the 14-year risk among HPV-negative women (overall; blue line) and the 9-year risk among HPV-negative women with a previous positive test (yellow line). In panel B the blue line is not visible due to the overlap with the red line. CIN2/3+, cervical intraepithelial neoplasia grade 2/3 or worse; HPV, human papillomavirus.

**

**

## **Fig B. Absolute risks of CIN3+ and CIN2+ amongst HPV-negative women randomized to the control trial group stratified for previous test result, after up to 3 screening rounds (14 years).**

Panels (A) to (C) refer to endpoint CIN3+ ((A) by previous HPV test, (B) by previous cytology test, (C) by previous co-test) and include a dotted line at 1% CIN3+ corresponding to the informal Dutch threshold for the next screening time-point; panels (D) to (F) refer to endpoint CIN2+ ((D) by previous HPV test, (E) by previous cytology test, (F) by previous co-test). Values embedded in the plots represent the 14-year risk among HPV-negative women (overall; blue line) and the 9-year risk among HPV-negative women with a previous positive test (yellow line). In panels B and E the blue line is not visible due to the overlap with the red line. CIN2/3+, cervical intraepithelial neoplasia grade 2/3 or worse; HPV, human papillomavirus.





# **Supplementary Tables**

## **Table A. Absolute risks of CIN3+ and CIN2+ at 14-year amongst HPV-negative women stratified for previous test result and age.**

|  |  | CIN3+ | | | CIN2+ | | |
| --- | --- | --- | --- | --- | --- | --- | --- |
|  | **N** | **N events** | **Risk % (95% CI)** | ***p*-value** | **N events** | **Risk % (95% CI)** | ***p*-value** |
| *Age <39 years* |  |  |  |  |  |  |  |
| Total | 2,331 | 18 | 0.95 (0.60 to 1.51) |  | 45 | 2.32 (1.73 to 3.10) |  |
| *Previous round* |  |  |  |  |  |  |  |
| HPV+ | 169 | 2 | 1.33 (0.33 to 5.25) | 0.613 | 6 | 4.30 (1.91 to 9.53) | 0.122 |
| HPV− | 2,160 | 16 | 0.91 (0.56 to 1.49) |  | 39 | 2.17 (1.58 to 2.96) |  |
| Cytology+ | 63 | 0 | 0.00 | - | 4 | 8.98 (3.25 to 23.51) | 0.008 |
| Cytology− | 2,268 | 18 | 0.97 (0.61 to 1.54) |  | 41 | 2.17 (1.60 to 2.94) |  |
| Co-test+ | 205 | 2 | 1.11 (0.27 to 4.39) | 0.801 | 7 | 4.22 (1.99 to 8.82) | 0.100 |
| Co-test− | 2,124 | 16 | 0.92 (0.57 to 1.51) |  | 38 | 2.14 (1.56 to 2.94) |  |
| *Age 39-48 years* |  |  |  |  |  |  |  |
| Total | 8,063 | 37 | 0.54 (0.39 to 0.74) |  | 78 | 1.17 (0.93 to 1.46) |  |
| *Previous round* |  |  |  |  |  |  |  |
| HPV+ | 222 | 6 | 3.50 (1.55 to 7.79) | <0.001 | 12 | 6.87 (3.89 to 11.98) | <0.001 |
| HPV− | 7,828 | 31 | 0.46 (0.32 to 0.66) |  | 66 | 1.02 (0.80 to 1.30) |  |
| Cytology+ | 180 | 1 | 0.69 (0.10 to 4.79) | 0.795 | 5 | 3.16 (1.32 to 7.45) | 0.024 |
| Cytology− | 7,883 | 36 | 0.53 (0.38 to 0.74) |  | 73 | 1.12 (0.89 to 1.41) |  |
| Co-test+ | 361 | 7 | 2.53 (1.19 to 5.34) | <0.001 | 15 | 5.30 (3.18 to 8.77) | <0.001 |
| Co-test− | 7,689 | 30 | 0.46 (0.32 to 0.65) |  | 63 | 0.99 (0.78 to 1.27) |  |
| *Age ≥49 years* |  |  |  |  |  |  |  |
| Total | 8,054 | 7 | 0.17 (0.07 to 0.40) |  | 24 | 0.62 (0.39 to 0.98) |  |
| *Previous round* |  |  |  |  |  |  |  |
| HPV+ | 121 | 1 | 0.83 (0.12 to 5.72) | 0.128 | 2 | 1.66 (0.42 to 6.47) | 0.167 |
| HPV− | 7,925 | 6 | 0.16 (0.07 to 0.40) |  | 22 | 0.60 (0.38 to 0.96) |  |
| Cytology+ | 170 | 0 | 0.00 | - | 4 | 2.76 (1.01 to 7.42) | 0.005 |
| Cytology− | 7,884 | 7 | 0.17 (0.08 to 0.41) |  | 20 | 0.57 (0.35 to 0.93) |  |
| Co-test+ | 268 | 1 | 0.37 (0.05 to 2.62) | 0.442 | 6 | 2.50 (1.11 to 5.61) | 0.002 |
| Co-test− | 7,778 | 6 | 0.16 (0.07 to 0.40) |  | 18 | 0.55 (0.33 to 0.92) |  |

CI, confidence interval; CIN2/3+, cervical intraepithelial neoplasia grade 2/3 or worse; HPV, human papillomavirus.

## **Table B. Absolute risks of CIN3+ and CIN2+ at 14-year amongst HPV-negative women stratified for previous test result, when the last preceding test result is used for stratification (sensitivity analysis).**

|  |  | CIN3+ | | | CIN2+ | | |
| --- | --- | --- | --- | --- | --- | --- | --- |
|  | **N** | **N events** | **Risk % (95% CI)** | ***p*-value** | **N events** | **Risk % (95% CI)** | ***p*-value** |
| *Pooled study groups* |  |  |  |  |  |  |  |
| Total | 18,448 | 62 | 0.48 (0.37 to 0.62) |  | 147 | 1.17 (0.99 to 1.38) |  |
| *Previous round* |  |  |  |  |  |  |  |
| HPV+ | 392 | 9 | 3.07 (1.56 to 5.98) | <0.001 | 18 | 6.02 (3.76 to 9.59) | <0.001 |
| HPV− | 18,035 | 53 | 0.43 (0.32 to 0.56) |  | 129 | 1.07 (0.89 to 1.27) |  |
| Cytology+ | 126 | 1 | 1.54 (0.22 to 10.42) | 0.244 | 7 | 7.73 (3.61 to 16.13) | <0.001 |
| Cytology− | 18,322 | 61 | 0.48 (0.37 to 0.62) |  | 140 | 1.13 (0.95 to 1.34) |  |
| Co-test+ | 479 | 9 | 2.65 (1.34 to 5.23) | <0.001 | 22 | 6.19 (4.02 to 9.47) | <0.001 |
| Co-test− | 17,948 | 53 | 0.43 (0.33 to 0.57) |  | 125 | 1.05 (0.87 to 1.25) |  |
| *Intervention group* |  |  |  |  |  |  |  |
| Total | 9,293 | 24 | 0.39 (0.26 to 0.59) |  | 65 | 1.05 (0.82 to 1.35) |  |
| *Previous round* |  |  |  |  |  |  |  |
| HPV+ | 145 | 4 | 3.99 (1.43 to 10.92) | <0.001 | 8 | 7.93 (3.87 to 15.90) | <0.001 |
| HPV− | 9,145 | 20 | 0.34 (0.22 to 0.53) |  | 57 | 0.95 (0.73 to 1.24) |  |
| Cytology+ | 73 | 1 | 2.56 (0.37 to 16.84) | 0.059 | 4 | 7.73 (2.84 to 20.10) | <0.001 |
| Cytology− | 9,220 | 23 | 0.38 (0.25 to 0.58) |  | 61 | 1.01 (0.78 to 1.30) |  |
| Co-test+ | 196 | 4 | 3.11 (1.09 to 8.71) | <0.001 | 11 | 7.95 (4.28 to 14.50) | <0.001 |
| Co-test− | 9,094 | 20 | 0.34 (0.22 to 0.54) |  | 54 | 0.92 (0.70 to 1.21) |  |
| *Control group* |  |  |  |  |  |  |  |
| Total | 9,155 | 38 | 0.57 (0.41 to 0.79) |  | 82 | 1.29 (1.03 to 1.61) |  |
| *Previous round* |  |  |  |  |  |  |  |
| HPV+ | 247 | 5 | 2.58 (1.05 to 6.26) | 0.001 | 10 | 5.05 (2.68 to 9.39) | <0.001 |
| HPV− | 8,890 | 33 | 0.52 (0.36 to 0.73) |  | 72 | 1.19 (0.94 to 1.50) |  |
| Cytology+ | 53 | 0 | 0.0 | - | 3 | 7.69 (2.36 to 23.51) | 0.003 |
| Cytology− | 9,102 | 38 | 0.57 (0.41 to 0.80) |  | 79 | 1.26 (1.00 to 1.58) |  |
| Co-test+ | 283 | 5 | 2.37 (0.95 to 5.82) | 0.002 | 11 | 5.08 (2.77 to 9.24) | <0.001 |
| Co-test− | 8,854 | 33 | 0.52 (0.37 to 0.74) |  | 71 | 1.18 (0.93 to 1.49) |  |

CI, confidence interval; CIN2/3+, cervical intraepithelial neoplasia grade 2/3 or worse; HPV, human papillomavirus.
