# Supplementary material for: Risk of cervical precancer among HPV–negative women in the Netherlands and its association with previous HPV and cytology results: A follow-up analysis of a randomized screening study
Source: PLoS Med. 2022 Oct 28;19(10):e1004115. doi: 10.1371/journal.pmed.1004115 (PMC9616214; doi:10.1371/journal.pmed.1004115)
Supplement: S2 File — (DOCX) [file pmed.1004115.s002.docx]

**S2 File: STROBE Checklist**

Supplement to:

**Risk of cervical precancer among HPV-negative women in the Netherlands and its association with previous HPV and cytology results: a follow-up analysis of a randomized screening study**

Federica Inturrisi, Lawrence Rozendaal, Nienke J. Veldhuijzen, Daniëlle A.M. Heideman, Chris J.L.M. Meijer, Johannes Berkhof

STROBE Statement: checklist of items that should be included in reports of observational studies.

|  | | Item No | Recommendation | |  | Section name |
| --- | --- | --- | --- | --- | --- | --- |
| **Title and abstract** | | 1 | (*a*) Indicate the study’s design with a commonly used term in the title or the abstract | |  | First page, Title |
|  |  |  | (*b*) Provide in the abstract an informative and balanced summary of what was done and what was found | |  | First page, Abstract |
| Introduction | | | | |  |  |
| Background/rationale | | 2 | Explain the scientific background and rationale for the investigation being reported | |  | Introduction, paragraphs 1/2 |
| Objectives | | 3 | State specific objectives, including any prespecified hypotheses | |  | Introduction, paragraph 3 |
| Methods | | | | |  |  |
| Study design | | 4 | Present key elements of study design early in the paper | |  | Methods, Study population paragraph 1 |
| Setting | | 5 | Describe the setting, locations, and relevant dates, including periods of recruitment, exposure, follow-up, and data collection | |  | Methods, Study population paragraphs 1/2 |
| Participants | | 6 | (*a*) *Cohort study*—Give the eligibility criteria, and the sources and methods of selection of participants. Describe methods of follow-up  *Case-control study*—Give the eligibility criteria, and the sources and methods of case ascertainment and control selection. Give the rationale for the choice of cases and controls  *Cross-sectional study*—Give the eligibility criteria, and the sources and methods of selection of participants | |  | Methods, Study population paragraphs 1/2, Statistical analyses |
|  |  |  | (*b*) *Cohort study*—For matched studies, give matching criteria and number of exposed and unexposed  *Case-control study*—For matched studies, give matching criteria and the number of controls per case | |  |  |
| Variables | | 7 | Clearly define all outcomes, exposures, predictors, potential confounders, and effect modifiers. Give diagnostic criteria, if applicable | |  | Methods, Statistical analyses |
| Data sources/ measurement | | 8* | For each variable of interest, give sources of data and details of methods of assessment (measurement). Describe comparability of assessment methods if there is more than one group | |  | Methods, Statistical analyses |
| Bias | | 9 | Describe any efforts to address potential sources of bias | |  | Methods, Statistical analyses |
| Study size | | 10 | Explain how the study size was arrived at | |  | Methods, Study population |
| Quantitative variables | | 11 | Explain how quantitative variables were handled in the analyses. If applicable, describe which groupings were chosen and why | |  | Methods, Statistical analyses |
| Statistical methods | | 12 | (*a*) Describe all statistical methods, including those used to control for confounding | |  | Methods, Statistical analyses |
|  |  |  | (*b*) Describe any methods used to examine subgroups and interactions | |  | Methods, Statistical analyses |
|  |  |  | (*c*) Explain how missing data were addressed | |  | Methods, Statistical analyses |
|  |  |  | (*d*) *Cohort study*—If applicable, explain how loss to follow-up was addressed  *Case-control study*—If applicable, explain how matching of cases and controls was addressed  *Cross-sectional study*—If applicable, describe analytical methods taking account of sampling strategy | |  | Methods, Statistical analyses |
|  |  |  | (*e*) Describe any sensitivity analyses | |  | Methods, Statistical analyses |
| Results | | | |  | | |
| Participants | 13* | (a) Report numbers of individuals at each stage of study—eg numbers potentially eligible, examined for eligibility, confirmed eligible, included in the study, completing follow-up, and analysed | | Results, paragraph 1, Fig 1 | | |
|  |  | (b) Give reasons for non-participation at each stage | | Fig 1 | | |
|  |  | (c) Consider use of a flow diagram | | Fig 1 | | |
| Descriptive data | 14* | (a) Give characteristics of study participants (eg demographic, clinical, social) and information on exposures and potential confounders | | Results, paragraph 1, Table 1 | | |
|  |  | (b) Indicate number of participants with missing data for each variable of interest | | Table 1 | | |
|  |  | (c) *Cohort study*—Summarise follow-up time (eg, average and total amount) | | Results, paragraph 2 | | |
| Outcome data | 15* | *Cohort study*—Report numbers of outcome events or summary measures over time | | Results, paragraph 2 | | |
|  |  | *Case-control study—*Report numbers in each exposure category, or summary measures of exposure | |  | | |
|  |  | *Cross-sectional study—*Report numbers of outcome events or summary measures | |  | | |
| Main results | 16 | (*a*) Give unadjusted estimates and, if applicable, confounder-adjusted estimates and their precision (eg, 95% confidence interval). Make clear which confounders were adjusted for and why they were included | | Results, paragraphs 2/7 | | |
|  |  | (*b*) Report category boundaries when continuous variables were categorized | |  | | |
|  |  | (*c*) If relevant, consider translating estimates of relative risk into absolute risk for a meaningful time period | | Results, paragraphs 2/7 | | |
| Other analyses | 17 | Report other analyses done—eg analyses of subgroups and interactions, and sensitivity analyses | | Results, paragraphs 3 and 7 | | |
| Discussion | | | |  | | |
| Key results | 18 | Summarise key results with reference to study objectives | | Discussion, paragraph 1 | | |
| Limitations | 19 | Discuss limitations of the study, taking into account sources of potential bias or imprecision. Discuss both direction and magnitude of any potential bias | | Discussion, paragraph 2 | | |
| Interpretation | 20 | Give a cautious overall interpretation of results considering objectives, limitations, multiplicity of analyses, results from similar studies, and other relevant evidence | | Discussion, paragraphs 3/4 | | |
| Generalisability | 21 | Discuss the generalisability (external validity) of the study results | | Discussion, paragraph 2 | | |
| Other information | | | |  | | |
| Funding | 22 | Give the source of funding and the role of the funders for the present study and, if applicable, for the original study on which the present article is based | | Funding statement | | |
